# Assafœtida in Hooping-Cough

**Published:** 1843-07

**Authors:** 


*Assafœtida in Hooping Cough.*—Dr. Reiken has derived more advantage from the use of assafœtida than from any other remedy in three epidemics of hooping-cough. It is indicated only after the febrile period has passed, and its influence is diminished in the third stage, when tonics should be combined with it. As assafœtida is a medicine which it would be very difficult to induce children to take by the mouth, Dr. Rieken prescribes it in enemas—he orders three grains of the gum resin to be rubbed up with a sufficient quantity of yolk of egg, and mixed with 125 to 250 scruples of water: This mixture will suffice for ten or twelve lavements for children under a year old; for from four to six, for children three years old; and for two or three only, for those that are older. For the first five or six days, two enemata are given daily, sometimes more; after that, one at night will be sufficient. If diarrhœa should come on, it may be stopped by increasing the quantity of the yolk, and by adding starch or gum arabic; olive oil should be added if tenesmus should supervene. The use of the assafœtida is very well borne by children of a strong constitution, and still better by those that are nervous or lymphatic.—*Annales de la Soc. Med. de Bruxelles*, from *Provincial Medical and Surgical Journal*, May 7, 1842.

